# Electroconvulsive therapy in a patient with resistant paranoid schizophrenia treated with clozapine

**DOI:** 10.1192/j.eurpsy.2021.2142

**Published:** 2021-08-13

**Authors:** S. Castelao-Almodóvar, A. Pérez-Balaguer, T. Ponte, B. Estevez Peña, Y.D. Corres Fuentes, E. Gil-Benito, E.M. Suárez Del Río, L. Gayubo-Moreo, B. Sanz-Aranguez

**Affiliations:** Psychiatry, Hospital Universitario Puerta de Hierro de Majadahonda, Madrid, Spain

**Keywords:** Electroconvulsive therapy, schizophrénia, resistant schizophrénia, clozapine

## Abstract

**Introduction:**

One of the usual indications for Electroconvulsive Therapy (ECT) is Paranoid Schizophrenia (PS), being performed usually in cases resistant to antipsychotics.

**Objectives:**

To present a clinical case of a patient with antipsychotic-resistant PS, including Clozapine, who received ECT.

**Methods:**

We present the case of a 47-year-old patient with an 8-years diagnosis of PS. He presented visual, auditory, and kinesthetic hallucinations, delusions, and thought insertion and diffusion phenomena that impeded concentration. He had received treatment with different antipsychotics (including Clozapine), without achieving remission of symptoms. He also presented significant adverse effects such as hypersalivation and extrapyramidal symptoms. Due to the poor response and the adverse effects that limited the dose increase, it was decided to start ECT.

**Results:**

The patient received a total of 9 sessions, presenting a significant reduction in symptoms since the 5th session (disappearance of the sensory-perceptual alterations and thought disturbances). As side effects, the patient presented amnesia of the moments prior to applying the therapy, which subsequently resolved. The patient continued to present concentration difficulties, although after ECT he denied the presence of thought insertion or diffusion phenomena to which he previously attributed the cause of these difficulties.

**Conclusions:**

Although less responsive than in other indications, ECT combined with antipsychotic drugs has been proven to be more effective than monotherapy (regardless of whether it’s Clozapine or another). This lower response could be due to the use of ECT in the most resistant cases, since it has been demostrated that in more acute cases a faster improvement occurs when the two treatments are combined.

**Disclosure:**

No significant relationships.

